# Support Vector Machine-based method for predicting subcellular localization of mycobacterial proteins using evolutionary information and motifs

**DOI:** 10.1186/1471-2105-8-337

**Published:** 2007-09-13

**Authors:** Mamoon Rashid, Sudipto Saha, Gajendra PS Raghava

**Affiliations:** 1 Bioinformatics Centre, Institute of Microbial Technology, Sector-39A, Chandigarh, India

## Abstract

**Background:**

In past number of methods have been developed for predicting subcellular location of eukaryotic, prokaryotic (Gram-negative and Gram-positive bacteria) and human proteins but no method has been developed for mycobacterial proteins which may represent repertoire of potent immunogens of this dreaded pathogen. In this study, attempt has been made to develop method for predicting subcellular location of mycobacterial proteins.

**Results:**

The models were trained and tested on 852 mycobacterial proteins and evaluated using five-fold cross-validation technique. First SVM (Support Vector Machine) model was developed using amino acid composition and overall accuracy of 82.51% was achieved with average accuracy (mean of class-wise accuracy) of 68.47%. In order to utilize evolutionary information, a SVM model was developed using PSSM (Position-Specific Scoring Matrix) profiles obtained from PSI-BLAST (Position-Specific Iterated BLAST) and overall accuracy achieved was of 86.62% with average accuracy of 73.71%. In addition, HMM (Hidden Markov Model), MEME/MAST (Multiple Em for Motif Elicitation/Motif Alignment and Search Tool) and hybrid model that combined two or more models were also developed. We achieved maximum overall accuracy of 86.8% with average accuracy of 89.00% using combination of PSSM based SVM model and MEME/MAST. Performance of our method was compared with that of the existing methods developed for predicting subcellular locations of Gram-positive bacterial proteins.

**Conclusion:**

A highly accurate method has been developed for predicting subcellular location of mycobacterial proteins. This method also predicts very important class of proteins that is membrane-attached proteins. This method will be useful in annotating newly sequenced or hypothetical mycobacterial proteins. Based on above study, a freely accessible web server TBpred http://www.imtech.res.in/raghava/tbpred/ has been developed.

## Background

According to the GOLD (Genomes OnLine Database) database [1] as on 12th Dec, 2006 genomes of nine mycobacterial species have been sequenced and published creating a heap of about 45055 kb of genomic data. The coming years will see a lot more as genome-sequencing projects are holding about 19 mycobacterial species in pipeline. Moreover, functions of 48% of the predicted 3995 proteins of *Mycobacterium tuberculosis *H37Rv are yet to be assigned [2]. Therefore a robust and reliable computer algorithm for functional annotation of mycobacterial proteins is the demand of time. This group of organism is well known for its pathogenicity. After Bacille Calmette-Guérin (BCG), developed in 1921, till date we don't have a promising vaccine against tuberculosis. Furthermore, several new pharmaceutical targets have yet to be unravelled to combat the multi-drug resistant strains of mycobacterium. One of the key features of Gene Ontology (GO) is cellular localization which gives important information about a protein [3,4]. Thus it is important to develop method for predicting subcellular localization of a protein of a pathogenic organism like mycobacterium.

In last few years several subcellular localization prediction systems have been developed using various features of a protein like composition of amino acid, pseudo amino acid, dipeptide and Physico-chemical properties [5-9]. Recently, a web server 'PseAA' [10] has been developed for computing pseudo amino acid composition, an important descriptor for protein sequence. Multiple alignments in form of PSSM profile have also been used to extract the compositional information for developing subcellular localization methods [11,12]. In these methods firstly a protein sequence is represented by fixed length pattern then models are developed using machine learning techniques like Support Vector Machine (SVM), Artificial Neural Network (ANN), K-nearest neighbor (KNN) [13-15]. Broadly, the existing methods of subcellular localization have been developed for i) eukaryotic proteins that includes TSSub, LOCSVMPSI, ESLpred, Euk-Ploc and BaCelLo [11,12,15-17] and ii) prokaryotic proteins mainly for bacterial proteins like PSORTb, PSLpred, CELLO, LOCtree, P-classifier, Gpos-ploc, GNBSL [18-26]. Recently, it has been observed that organism specific method performs better than general methods for that organism [13,27-29]. Thus methods have been developed for predicting subcellular location of human proteins [13,27,29]. One of the challenges in subcellular localization is to predict location of proteins having multiple-location [29,30]. Other subcellular location predictors have been developed very recently for a wide variety of organism type such as plant, bacteria and virus [31-33]. In addition attempts have been made to annotate *Mycobacterium tuberculosis *genome using experimental and predicted information [34,35].

To the best of authors' knowledge no method has been developed for predicting subcellular localization of mycobacterial proteins, which has different cell wall composition than Gram-negative or Gram-positive bacteria. In this study we describe models developed for predicting four subcellular locations of mycobacterial proteins, namely cytoplasmic, Integral membrane, secretory and membrane-attached proteins [36,37]. A systematic attempt has been made to develop highly accurate SVM-based models using various features of proteins like amino acid, dipeptides and PSSM composition [38,39]. In addition models have been developed using Hidden Markov Model (HMM) and MEME/MAST for predicting subcellular location of mycobacterial proteins [40-43]. We also compared performance of our method with that of the other existing methods on dataset used in the current study.

## Results

### Performance of BLAST

Biologists routinely use the BLAST for similarity search; it will be interesting to know the performance of BLAST on the same dataset and cross-validation used in this study. This is important because users wish to know the advantage of any sophisticated method over routinely used method like BLAST. Thus we evaluated performance of BLAST on our dataset using five-fold cross validation where proteins in test dataset were searched using BLAST against proteins of training dataset. As shown in Table 1, the performance of BLAST is very low except for secretory proteins. The detailed statistic has been included in Table S1, Additional file 1. These results show that our training and testing datasets are non-redundant.

**Table 1:** Prediction of subcellular localization of proteins using BLAST

	Cytoplasmic		Integral Membrane		Secretory		Membrane -attached	
	
BLAST E-value	Prob	Acc	Prob	Acc	Prob	Acc	Prob	Acc
**1E-004**	20.0	0.6	0.0	0.0	86.9	40.0	0.0	0.0
**1E-003**	21.4	0.9	0.0	0.0	86.9	40.0	0.0	0.0
**1E-002**	55.0	6.5	33.3	1.2	80.0	40.0	16.6	1.7
**1E-001**	50.0	12.1	70.1	13.4	68.9	40.0	16.6	1.7
**1**	46.7	29.4	72.2	41.3	51.3	40.0	8.6	5.0
**10**	42.3	41.8	69.0	65.9	41.6	40.0	20.7	20.0
**100**	45.6	45.6	70.5	70.2	40.0	40.0	23.3	23.3
**1000**	46.5	46.5	70.4	70.4	40.0	40.0	26.7	26.7

Prob: Probability of correct hit; Acc: Accuracy

### SVM Models

The performance of all the modules developed in this study was evaluated using 5-fold cross-validation technique. The performances of different SVM modules have been summarized in Table 2. We achieved maximum overall accuracy of 82.51% using amino acid composition based SVM model (kernel = RBF, ϒ = 0.1 and C = 600). Though overall accuracy was 82.51%, average accuracy was only 68.47%. It is because the method performed well on cytoplasmic and integral membrane protein but performed poorly on the remaining two classes (secretory and membrane-attached). As numbers of proteins in cytoplasmic and integral membrane classes were much higher than remaining two classes so, overall accuracy was higher. We also developed SVM model using dipeptides composition and attained maximum overall accuracy of 80.39%, which is slightly lower than that achieved by amino acid composition based method. It has been shown in the past that evolutionary information obtained from protein sequence provides more insight than protein sequence per se. Thus SVM based model has been developed using evolutionary information extracted from PSSM profile of PSI-BLAST. This model performed better than amino acid and dipeptide composition based models and achieved maximum overall accuracy of 86.62% and average accuracy of 73.71%. This demonstrates the advantage of evolutionary information in prediction of subcellular location of proteins. The SVM based models failed to predict secretory and membrane-attached proteins with high accuracy; it may be due to lower number of proteins in these classes. This is a major limitation of machine learning techniques that their performance is biased by number of proteins in a class used for training. The performance of polynomial and linear kernels using PSSM is shown in Table S2, Additional file 1.

**Table 2:** The performance of various SVM models

	Cytoplasmic		Integral Membrane		Secretory		Membrane-attached		Overall accuracy	Average accuracy
	
Input Pattern	ACC ± sd	MCC	ACC ± sd	MCC	ACC ± sd	MCC	ACC ± sd	MCC	ACC	ACC
**Amino Acid Composition**	88.82 ± 5.4	0.77	86.07 ± 7.5	0.71	44.00 ± 42.2	0.57	55.00 ± 19.4	0.58	82.51	68.47
**Dipeptide Composition**	89.41 ± 7.8	0.72	81.09 ± 7.5	0.67	50.00 ± 36.8	0.60	50.00 ± 17.4	0.57	80.39	67.63
**PSSM profile**	94.71 ± 4.8	0.85	87.81 ± 6.1	0.80	44.00 ± 42.2	0.48	68.33 ± 28	0.69	86.62	73.71

ACC: Accuracy; MCC: Matthews correlation coefficient; sd: Standard Deviation

### HMM Profile

In this study, HMM based models have been developed for each subcellular location. The performance of HMM models for each class/location is shown in Table 3. As shown in Table 3, HMM model performed well for secretory proteins where it predicted 38% secretory proteins at E-value 1E-003, whereas for other classes it had poor hits. At E-value 1E-001, the percent of correct hits for cytoplasmic, integral membrane, secretory and membrane-attached proteins were 20.59, 21.39, 40.00 and 65.00 respectively. Though percent of hits increases with higher E-value but at the same time it also elevates number of false hits. Thus overall performance of HMM based models alone or in combination with SVM models was poor (data not shown).

**Table 3:** The performance of HMM based model

	Sensitivity (percent of correct hits)					
	
E-value	Cytoplasmic	Integral Membrane	Secretory	Membrane -attached	Overall accuracy	Average accuracy
**1E-003**	0.29	1.99	38.00	5.00	3.63	11.32
**1E-002**	3.82	5.47	38.00	41.67	9.26	22.24
**1E-001**	20.59	21.39	40.00	65.00	25.23	36.74
**1**	33.82	30.60	40.00	70.00	35.21	43.60
**10**	36.18	32.84	40.00	73.33	37.44	45.58
**20**	36.18	32.84	40.00	73.33	37.44	45.58

### MEME/MAST Motif

The HMM based method allows performing similarity search at sequence level but not at motif level. Thus in this study motifs were extracted and searched using MEME/MAST software. As shown in Table 4 (parentheses) and Table S3-S6, Additional file 1, the MEME/MAST motif-based models performed well for secretory and membrane-attached proteins but failed for cytoplasmic and integral membrane proteins. These results suggested that motif-based approach alone was not sufficient for predicting all subcellular locations. Moreover, it was interesting to note that SVM models failed to predict these two classes of proteins (secretory and membrane-attached) with a fair accuracy.

**Table 4:** The comparison of performance of hybrid model and MEME/MAST model

	Percent accuracy					
	
E-value	Cytoplasmic	Integral Membrane	Secretory	Membrane -attached	Overall accuracy	Average accuracy
**1E-003**	94.7 (0.0)	87.8 (0.2)	46.0 (40.0)	65.0 (0.0)	86.5 (2.4)	73.4 (10.1)
**1E-002**	94.7 (0.0)	87.6 (1.5)	46.0 (40.0)	65.0 (0.0)	86.3 (3.0)	73.4 (10.4)
**1E-001**	94.7 (0.0)	87.3 (2.7)	46.0 (40.0)	65.0 (0.0)	86.2 (3.6)	73.3 (10.7)
**1**	93.2 (0.3)	86.6 (8.5)	46.0 (40.0)	65.0 (11.7)	85.3 (7.3)	73.2 (15.1)
**10**	**87.0 (0.9)**	**85.3 (22.4)**	**92.0 (100.0)**	**91.7 (91.7)**	**86.8 (23.2)**	**89.0 (53.4)**
**20**	79.1 (0.9)	85.3 (31.6)	92.0 (100.0)	91.7 (100.0)	83.7 (28.1)	87.0 (58.1)
**30**	75.0 (4.4)	84.3 (39.1)	92.0 (100.0)	91.7 (100.0)	81.6 (33.1)	85.8 (60.9)

### Hybrid Approach

As shown above in Table 2 and Table 4 (parentheses), SVM models performed well on cytoplasmic and integral membrane where as MEME/MAST motif models performed well on secretory and membrane-attached proteins. Thus there was a need to combine these models in order to develop a highly accurate approach. So a hybrid model was developed where a protein is predicted using SVM and MEME/MAST motif with preference given to MEME/MAST motif. In hybrid model first a protein sequence was searched against all the motifs, if any motif has E-value lower than cut-off value then motif location is assigned as location of protein. In case more than one motif is found in protein then location of motif having minimum E-value is assigned as location of a protein. In case protein does not have any motif then PSSM based SVM models are used to predict its subcellular location. For detailed scheme see Table S7 in Additional File 1. As shown in Table 4, we achieved best performance at E-value 10 with overall accuracy of 86.8%. Though the overall performance was not very high as compared to PSSM based SVM model but average accuracy increases around 16% (from 73.71 to 89%). It means performances for all classes were higher, rather than for only cytoplasm and integral membrane protein.

### Reliability Index

**Figure 1:**
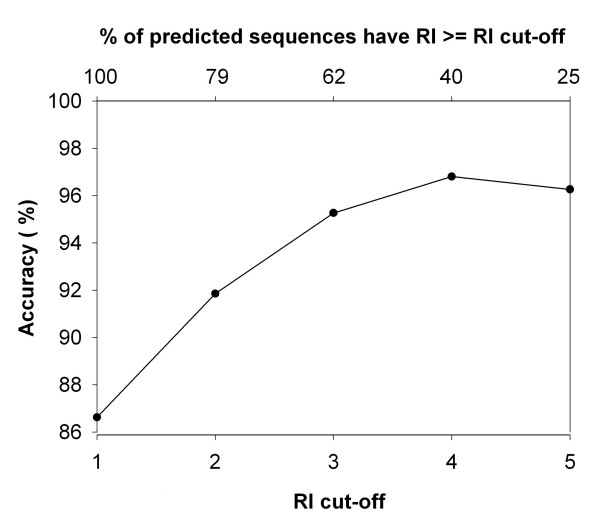
A plot between reliability index (RI) and percent coverage vs average accuracy for PSSM based SVM module, where Y-axis shows average accuracy and X-axis shows RI (lower axis) and percent coverage (upper axis). For example, about 62% of sequences having RI > = 3 are predicted with 95% accuracy.

In order to provide confidence in prediction, we computed reliability index (RI). It is a measure of level of certainty in a prediction. Figure 1 shows the average prediction accuracy with reliability index greater than or equal to a given value n where n = 1, 2, 3, 4 and 5. About 62% of the sequences with RI > = 3 are predicted with 95% accuracy by our PSSM based SVM module. The RI plots of amino acid composition and dipeptides composition based SVM modules are available in Additional File 1, Figure S1 and Figure S2 respectively.

### Comparison with existing methods

It is important to compare newly developed method with existing methods. This is the first subcellular location prediction method for mycobacterial proteins, thus it is difficult to compare this method with existing methods. It is known that mycobacterium species are significantly similar to Gram-positive bacteria, thus we compare our method with existing methods developed for Gram-positive bacteria. One to one comparison of existing method with our method was not possible because number of subcellular locations predicted by these methods was different than locations predicted by our method. For example none of the existing methods predicts membrane-attached proteins. In order to provide comprehensive comparison, existing methods have been evaluated on the dataset used in this study and presented by confusion matrix (Table 5). First proteins were predicted using PSORTb version 2.0, it correctly predicted 88% cytoplasmic, 81% integral membrane and 80% secretory proteins. PSORTb predicted only 18% membrane-attached into cytoplasmic membrane proteins and rest of them as unknown proteins. We predicted subcellular location of proteins using Proteome Analyst Specialized Subcellular Localization Server v2.5 (PA-SUB), it correctly predicted 95% cytoplasmic and 100% secretory protein. Surprisingly it predicts integral membrane protein either as extracellular or "no-positive prediction" instead of predicting as plasma membrane protein. Only 17% membrane-attached proteins were predicted as plasma membrane protein. In this study, we considered only top prediction if PA-SUB predicts more than one location for a protein. We were unable to evaluate, recently developed Gpos-PLoc method (trained on Gram- positive bacterial proteins) because it predicted subcellular location of one protein at a time. In addition we also evaluated TMHMM which is a specialized method for predicting membrane proteins. As shown in Table 5, it correctly predicted 88% integral membrane proteins as membrane proteins. Like other methods it also failed to predict membrane-attached proteins. These comparisons show our method performs better than any of the existing programs on Gram-positive bacterial proteins.

**Table 5:** The performances of existing methods on dataset used in this study

Methods	Predicted Locations	Cytoplasmic [340]	Intergral Membrane [402]	Secretory [50]	Membrane -attached [60]
**PSORTB**	Cytoplasm	300 (88%)	20	3	4
	Extracellular	2	4	40 (80%)	4
	Cytoplasmic Membrane	3	326 (81%)	0	11 (18%)
	Cell Wall	0	1	0	1
	Unknown	35	51	7	40
**PA-SUB**	Cytoplasm	323 (95%)	31	0	0
	Extracellular	1	117	50 (100%)	50
	Plasma Membrane	1	0 (0%)	0	10 (17%)
	No-positive	15	254	0	0
**TMHMM**	Membrane	1	354 (88%)	40	14 (23%)
	Non-Membrane	339	48	10	46

### Web server description

Various SVM modules developed in the present study were implemented into a web server, TBpred, for predicting the subcellular localization of mycobacterial proteins. User can select from amino acid composition, dipeptide composition and PSSM based SVM models or a hybrid model for prediction. The common gateway interface (CGI) script for TBpred was written using PERL 5.03. This server is installed on a Sun Server (420E) under a UNIX (Solaris 7) environment. TBpred is freely available at http://www.imtech.res.in/raghava/tbpred/.

## Discussion

Several methods have been developed for predicting subcellular location of eukaryotic, prokaryotic (Gram-negative bacteria) and human proteins but no method is available for mycobacterial proteins. Thus there was a need to develop a dedicated method for predicting subcellular localization of mycobacterial proteins. There are two reasons for developing subcellular localization method specially for mycobacterial proteins; i) organism specific subcellular localization method(s) performs better than generalized methods [13,27-29]; ii) Mycobacterium sp. is different from other organisms (it has complex cell wall and its virulence factors are distinct from other pathogens). Thus we made systematic attempt to develop method for predicting subcellular localization of mycobacterial proteins using state of the art techniques. First standard SVM models have been developed using amino acid and dipeptides composition. The performance of these standard models was excellent for cytoplasmic and integral membrane proteins but failed to predict secretory and membrane-attached proteins (Table 2). The performance improved significantly from 68.47% to 73.71% when PSSM composition is used instead of amino acid composition. Despite overall improvement, accuracy of prediction was low for secretory proteins, though accuracy increased in case of membrane-attached proteins. The failure of these models for secretory and membrane-attached proteins may be due to two reasons-(1) small number of proteins in these locations used for training the model; (2) their amino acid composition is significantly different.

In order to overcome these limitations we developed HMM based models for predicting subcellular location. The performance of HMM based model was reasonable for secretory and membrane-attached proteins but its performance was poor for other two classes (Table 3). It seems that secretory and membrane-attached proteins have signals. We also combined HMM model with PSSM based SVM model but performance did not improve (data not shown). We also developed motif-based method using MEME/MAST, where MEME is used to discover motifs and MAST is used to search these motifs in protein database. As shown in Table 4 (parentheses), motif based model successfully predicted secretory proteins; it means secretory proteins have signals which are detected by MEME/MAST. The motif-based method also predicted membrane-attached proteins with reasonable accuracy, but it failed to predict other two classes' particularly cytoplasmic proteins. It is because cytoplasmic proteins are very different so they do not have any specific motifs. Membrane proteins maintain certain type of secondary structure so there may be few motifs in these proteins. It is concluded therefore that for subcellular localization prediction one approach is not sufficient. Most of the pre-existing methods were either based on composition or based on signal/motif, thus their performance was not high for all locations. It's important to combine two approaches in order to predict all subcellular location with high accuracy. The quest arose how to combine two approaches in order to use their strength. In motif based approaches probability of correct prediction depends on E-value. Thus, first we searched motifs in a protein using MAST, if it has motif then we assigned motif's location as protein's location. In case if protein has no motif then we predicted its location using PSSM based SVM model. The average accuracy increased around 17% with minimum accuracy of 85.3% for a particular location. We also compared our method with existing methods, though one to one comparison was not possible as locations were not same. The performance of our method was better than existing methods on our dataset. Our method predicts very important class of proteins called membrane-attached proteins [36].

## Conclusion

A new subcellular class of mycobacterial proteins named "membrane-attached by lipid anchor" has been introduced for the first time. This class of protein may play a role in enhancing the immune response of the host by acting as surface antigens. Thus the search for a potential vaccine/drug target for this immensely important bacterial pathogen by the experimental researchers will greatly be appended by the prediction algorithm developed in this study. Moreover, the comparison of TBpred prediction efficiency with existing methods developed for Gram- positive bacteria supported our earlier assumption that organism specific classifier performs better than the generalised one.

## Methods

### The Data Set

The mycobacterial protein sequences were extracted from release 48 of Swiss-Prot. Initially, we got 1365 mycobacterial proteins; after removing the fragments and the non-experimental qualifier "by similarity" we got 882 proteins. These 882 proteins belong to 13 subcellular locations as shown in Table 6. Among 13 different subcellular compartments, four major locations were selected containing reasonable number of proteins. The final dataset had total 852 proteins with 340 cytoplasmic, 402 integral membranes, 50 secretory and 60 proteins attached to the membrane by a lipid anchor.

**Table 6:** Statistics of distributions of proteins among different subcellular locations

Subcellular localizations	Sample Numbers
1. Probably external side of the cell wall	1
2. Integral membrane protein	402
3. Cytoplasmic	340
4. Secreted	50
5. Membrane associated	10
6. Soluble or peripheral membrane protein	3
7. Attached to the membrane by a lipid anchor	60
8. Probable peripheral membrane protein	3
9. Type-I membrane protein	2
10. Surface associated	2
11. Membrane bound	5
12. Membrane protein	3
13. Partially secreted	1

**Total**	**882**

### Non-redundant dataset

Ideally one should remove similar proteins from data in order to generate the non-redundant data, as similar protein in training and testing data influence the performance of a method. We computed level of similarity in proteins belonging to a subcellular location using CD-HIT. As shown in Table 7, number of proteins remaining in different locations reduced significantly when we remove redundant proteins at 40% identity. Thus it was not a wise step to remove redundant proteins from our dataset as it reduced the size significantly. In order to overcome this problem, we used the BLAST-clustering approach earlier adopted by [37]. In BLAST-cluster approach, clusters are created for each location of proteins in such a way that no protein in any cluster has sequence similarity more than e-value 10 e-4 (around 26% percent identity) with any protein of other clusters. These clusters are used to create five sets in such a way that protein in one set does not have similarity with proteins in other remaining sets, though proteins in the same set may be redundant. These way non-redundant sets were created without removing any protein from the dataset.

**Table 7:** Number of proteins remaining in various locations, after removing redundant proteins, at cut-off 40%, 60% and 90% using program CD-HIT

	Sequences remaining after removal of similar sequences			
	
CD-HIT cut-off (% identity)	Cytoplasmic (340*)	Integral-membrane (402)	Secretory (50)	Membrane-attached (60)
**90**	223	262	34	38
**60**	118	195	20	29
**40**	117	182	17	27

* Number in bracket is total number of proteins in a location

### Five-fold cross validation

Ideally one should evaluate newly developed method using jack-knife method (leave one out cross-validation) [44,45]. In jack-knife test each protein is used for testing and remaining proteins are used for training, it means one should repeat the process N times for N number of proteins. But in practice limited cross-validation technique (like five-fold, seven-fold) is commonly used instead of jack-knife [46-48]. In this study we evaluated all models using five-fold cross-validation technique, where dataset is randomly divided into five sets, and each containing equal number of proteins. Four sets are used for training and remaining one set for testing; this process is repeated five times in such a way that each set is used once for testing. Finally average of five sets is calculated.

### Support Vector Machine Models

In this study, Support Vector Machine has been implemented using SVM^light^, which is widely used for developing methods in the field of bioinformatics [38,45-51]. We used SVM^light ^binary classifier using 1-*vs-*r (one-versus-rest) approach, for developing model for predicting multiple locations. In this 1-*vs*-r approach a SVM model was built for each class by considering proteins of that class positive and proteins of rest of the classes as negative.

### Amino Acid and Dipeptide Composition

The percent amino acid composition of each amino acid was calculated using standard formula described in the past [20]. These compositions are represented by a vector of dimension 20. Similarly dipeptide composition of a protein was calculated and represented by a vector of dimension 400 [49,50].

### Composition of Position-Specific Scoring Matrix

The PSSM profile for each protein was generated using PSI-BLAST [39] by searching the protein against NR database obtained from NCBI. The PSI-BLAST was used with cut-off value 0.001 with three iterations. The PSSM scores were normalized in order to get values between 0 and 1, and then position specific composition of each amino acid was calculated. This way we got composition of amino acids with evolutionary information in form of 400 values [12].

### HMM Profiles

HMM turns a multiple sequence alignment into a position specific searching system suitable for searching databases for remotely homologous sequences. HMM analysis complements standard pair wise comparison method for large-scale sequence analysis [40]. HMM profiles were generated using software HMMER V-2.3.2. Sean Eddy at Washington University developed HMMER [41].

### Multiple Em for Motif Elicitation/Motif alignment and Search Tool (MEME/MAST)

Motif is a pattern of nucleotides or amino acids that appear in a DNA or protein family. The MEME/MAST consists of two programs, one allows discovery of motifs shared by closely related sequences (MEME) [42] and the other facilitates database search for sequences containing these motifs (MAST) [43]. Motifs in related protein sequences occur not merely by chance but because they share some biological functions. These motifs might be the active sites of related enzymes. In the present study meme-3.0.14 version is used. We conducted our study for each subcellular localization class independently keeping in mind that the proteins belonging to a subcellular localization class might share some subsequences and thus some biological functions. The motifs discovered in a subset of samples by MEME were searched within the sequences of another subset of the same family (considered as positive database) and also within the samples of rest of the classes (considered as negative database) by MAST. Hit from samples within the class and outside the class was used to evaluate the efficacy of the MEME/MAST classification system. Expectation value (E-value) cut-off was also taken into account during MAST analysis. If the hits for a protein sample were from both within the class and outside the class, hit with lower E-value was preferred.

### Hybrid module

We combined the output of MEME/MAST and the output SVM module. Firstly, a comprehensive list was generated that encapsulate the hits, along with their corresponding E-value, from all MEME/MAST model (cytoplasmic, integral membrane, secreted and membrane-attached) and the SVM prediction for each protein in the dataset. The MEME/MAST decision (if any) is given priority upon SVM prediction in the final assignment of class to a particular protein sample. In case if all MEME/MAST models generated hits for a sample, the sample was classified into the model generating hit with lowest E-value. Moreover if the lowest E-value is shared by more than one model (although it is the rare finding), the final decision was taken on consensus among MEME/MAST and SVM models. If MAST produces no hit at given E-value then SVM model was used to predict subcellular location of a protein.

### Performance Measures

The performance of all modules developed in this study was computed using standard parameters like accuracy (Acc) and Matthews correlation coefficient (MCC). Following equations were used to compute these parameters -

(1) $$Accuracy(x)=\frac{p(x)}{Exp(x)}$$

(2) $$MCC(x)=\frac{p(x)n(x)-u(x)o(x)}{\sqrt{\left(p(x)+u(x))(p(x)+o(x))(n(x)+u(x))(n(x)+o(x)\right)}}$$

Where *x *can be any subcellular location (nuclear, cytoplasm, extracellular and mitochondria), exp(x) is the number of sequences observed in location x, p(x) is the number of correctly predicted sequences of location x, n(x) is the number of correctly predicted sequences not of location x, u(x) is the number of under-predicted sequences and o(x) is the number of over-predicted sequences.

### Overall and average accuracy

In this study we computed both overall and average accuracy. The overall accuracy is the percent of correctly predicted proteins irrespective of class. The average accuracy is mean accuracy of four classes. Both type of accuracy have their advantage and disadvantage.

### Reliability Index (RI)

Reliability index is a simple indication of level of certainty in the prediction. The strategy followed to calculate the RI is similar to that mentioned by [8].

$$\text{RI}=\{\begin{array}{ll} \text{INT}(\text{diff}*5/3+1 & \text{if }0<=\text{diff}<4,\\ 5 & \text{if diff}>=4. \end{array}$$

Assignment of RI to each sequence is based upon the difference of highest and the second highest scores of various 1-v-r SVMs in the multi-class classification. RI is defined as:

## Authors' contributions

MR and SS created datasets, developed various modules and evaluated all modules. GPSR conceived the idea, coordinated it and refined the manuscript drafted by MR and SS. All the authors have read and approved the final manuscript.

## Supplementary Material

Additional file 1 Supplementary material. It consists of various tables and figures that were somehow supportive to the conducted study. Click here for file
