# Supplementary material for: Support Vector Machine-based method for predicting subcellular localization of mycobacterial proteins using evolutionary information and motifs
Source: BMC Bioinformatics. 2007 Sep 13;8:337. doi: 10.1186/1471-2105-8-337 (PMC2147037; doi:10.1186/1471-2105-8-337)
Supplement: Additional file 1 — Supplementary material. It consists of various tables and figures that were somehow supportive to the conducted study. [file 1471-2105-8-337-S1.pdf]

# Supplementary Material

## Support Vector Machine-based method for predicting subcellular localization of mycobacterial proteins using evolutionary information and motifs

Mamoon Rashid, Sudipto Saha and G. P. S. Raghava*
Institute of Microbial Technology, Sector-39A, Chandigarh, India

Running Title: **Subcellular localization of mycobacterial proteins**

Address correspondence to: Dr. G. P. S. Raghava, Scientist, Bioinformatics Centre Institute of Microbial Technology Sector 39A, Chandigarh, INDIA, Phone: +91-172-2690557; Fax: +91-172-2690632 E-mail: raghava@imtech.res.in

---

The data shown in this supplementary material are all produced during the course of study. The performance of BLAST for subcellular location prediction has been evaluated (Table S1). PSSM based SVM modules have been best optimized on Radial kernel with g=2, c=50 and j=1 giving rise to overall accuracy of 86.62%.Linear (c = 50, j = 5) and polynomial (d=1, c=50, j=5) kernels were also optimized to give overall accuracy of 85.3% and 85.3% respectively (Table S2). Performance of various MEME/MAST binary modules has been analyzed. Four different motifs based binary classification modules were developed for cytoplasmic, integral membrane, secretory and membrane attached classes (Table S3 – S6). The decision of assignment of a localization class to protein samples in hybrid module is shown in Table S7. The Table S7 shows seven columns. First column is for ID, 2nd to 5th for hits of MEME/MAST models for cytoplasmic, integral membrane, secretory and membrane attached protein classes respectively. 6th column for SVM prediction and last column states final decision. Final decision is taken according to meme models (if any hit is present). In absence of meme hits for any sample, SVM prediction is considered. If hit for a sample comes from more than one meme models the final decision will be the hit with lowest E-value.

**Table S1:** The comprehensive statistics showing performance of BLAST.

| E-value | Cytoplasmic [340] | | | | Integral membrane [402] | | | | Secretory [50] | | | | Membrane attached [60] | | | |
|---|---|---|---|---|---|---|---|---|---|---|---|---|---|---|---|---|
| | **H** | **C** | **P** | **A** | **H** | **C** | **P** | **A** | **H** | **C** | **P** | **A** | **H** | **C** | **P** | **A** |
| **0.0001** | 10 | 2 | 20.0 | 0.6 | 5 | 0 | 0.0 | 0.0 | 23 | 20 | 86.9 | 40.0 | 2 | 0 | 0.0 | 0.0 |
| **0.001** | 14 | 3 | 21.4 | 0.9 | 7 | 0 | 0.0 | 0.0 | 23 | 20 | 86.9 | 40.0 | 3 | 0 | 0.0 | 0.0 |
| **0.01** | 40 | 22 | 55.0 | 6.5 | 15 | 5 | 33.3 | 1.2 | 25 | 20 | 80.0 | 40.0 | 6 | 1 | 16.6 | 1.7 |
| **0.1** | 82 | 41 | 50.0 | 12.1 | 77 | 54 | 70.1 | 13.4 | 29 | 20 | 68.9 | 40.0 | 6 | 1 | 16.6 | 1.7 |
| **1** | 214 | 100 | 46.7 | 29.4 | 230 | 166 | 72.2 | 41.3 | 39 | 20 | 51.3 | 40.0 | 35 | 3 | 8.6 | 5.0 |
| **10** | 336 | 142 | 42.3 | 41.8 | 384 | 265 | 69.0 | 65.9 | 48 | 20 | 41.6 | 40.0 | 58 | 12 | 20.7 | 20.0 |
| **100** | 340 | 155 | 45.6 | 45.6 | 400 | 282 | 70.5 | 70.2 | 50 | 20 | 40.0 | 40.0 | 60 | 14 | 23.3 | 23.3 |
| **1000** | 340 | 158 | 46.5 | 46.5 | 402 | 283 | 70.4 | 70.4 | 50 | 20 | 40.0 | 40.0 | 60 | 16 | 26.7 | 26.7 |

Where H is Number of hits; C is Number of correct hits; P is percent of correct hit (C/H * 100) and A is percent accuracy (C/total number of proteins in the particular class *100)

**Table S2:** The performance of PSSM based SVM modules using different kernel functions at optimized parameters. The performance was evaluated by 5-fold cross-validation method.

| Sub-cellular Localization | Linear kernel | | Polynomial kernel | | Radial kernel | |
|---|---|---|---|---|---|---|
| | **ACC** | **MCC** | **ACC** | **MCC** | **ACC** | **MCC** |
| **Cytoplasmic** | 93.5 | 0.85 | 93.5 | 0.85 | 94.7 | 0.85 |
| **Integral membrane** | 88.6 | 0.76 | 88.6 | 0.76 | 87.8 | 0.8 |
| **Secretory** | 40 | 0.49 | 40 | 0.49 | 44 | 0.48 |
| **Membrane attached** | 55 | 0.58 | 55 | 0.58 | 68.3 | 0.69 |
| **Average** | **85.3** | **0.77** | **85.3** | **0.77** | **86.6** | **0.79** |

ACC: Accuracy; MCC: Matthews correlation coefficient

**Table S3:** Performance of cytoplasmic motif based study.

| E-value | Sensitivity | Specificity | Accuracy | MCC |
|---|---|---|---|---|
| **$10^{-3}$** | 0.0 | 100.0 | 60.1 | 0.00 |
| **$10^{-2}$** | 0.0 | 99.8 | 60.0 | 0.00 |
| **$10^{-1}$** | 0.0 | 99.6 | 59.9 | 0.00 |
| **$10^{0}$** | 0.3 | 99.6 | 60.0 | 0.00 |
| **10** | 0.9 | 98.6 | 59.6 | 0.00 |
| **20** | 0.9 | 97.7 | 59.0 | 0.00 |
| **30** | 4.4 | 96.9 | 60.0 | 0.03 |
| **40** | 8.5 | 94.9 | 60.4 | 0.06 |
| **50** | 17.1 | 93.9 | 63.3 | 0.18 |
| **60** | 32.4 | 92.2 | 68.3 | 0.32 |
| **70** | 100.0 | 91.8 | 95.1 | 0.90 |

**Table S4:** Performance of integral membrane protein motif based study.

| E-value | Sensitivity | Specificity | Accuracy | MCC |
|---|---|---|---|---|
| **$10^{-3}$** | 0.2 | 100 | 52.9 | 0.04 |
| **$10^{-2}$** | 1.5 | 100 | 53.5 | 0.09 |
| **$10^{-1}$** | 2.7 | 100 | 54.1 | 0.12 |
| **$10^{0}$** | 8.5 | 98.9 | 56.2 | 0.18 |
| **10** | 22.4 | 97.1 | 61.9 | 0.3 |
| **20** | 31.6 | 94 | 64.6 | 0.33 |
| **30** | 39.1 | 90.4 | 66.2 | 0.35 |
| **40** | 44 | 86.9 | 66.7 | 0.35 |
| **50** | 53.2 | 84.7 | 69.8 | 0.4 |
| **60** | 60.9 | 83.3 | 72.8 | 0.46 |
| **70** | 69.7 | 81.1 | 75.7 | 0.51 |

**Table S5:** Performance of secretory proteins motif based study.

| E-value | Sensitivity | Specificity | Accuracy | MCC |
|---|---|---|---|---|
| **$10^{-3}$** | 40.0 | 99.8 | 96.2 | 0.59 |
| **$10^{-2}$** | 40.0 | 99.8 | 96.2 | 0.59 |
| **$10^{-1}$** | 40.0 | 99.8 | 96.2 | 0.59 |
| **$10^{0}$** | 40.0 | 99.6 | 96.1 | 0.58 |
| **10** | 100.0 | 97.5 | 97.7 | 0.84 |
| **20** | 100.0 | 95.4 | 95.7 | 0.74 |
| **30** | 100.0 | 93.5 | 93.9 | 0.68 |
| **40** | 100.0 | 91.6 | 92.1 | 0.63 |
| **50** | 100.0 | 90.1 | 90.7 | 0.59 |
| **60** | 100.0 | 88.3 | 89.0 | 0.55 |
| **70** | 100.0 | 86.3 | 87.1 | 0.52 |

**Table S6:** Performance of membrane attached proteins motif based study.

| E-value | Sensitivity | Specificity | Accuracy | MCC |
|---|---|---|---|---|
| **$10^{-3}$** | 0.0 | 99.7 | 92.7 | 0.00 |
| **$10^{-2}$** | 0.0 | 99.7 | 92.7 | 0.00 |
| **$10^{-1}$** | 0.0 | 99.7 | 92.7 | 0.00 |
| **$10^{0}$** | 11.7 | 99.0 | 92.8 | 0.21 |
| **10** | 91.7 | 97.3 | 96.9 | 0.80 |
| **20** | 100.0 | 95.5 | 95.8 | 0.77 |
| **30** | 100.0 | 94.2 | 94.6 | 0.73 |
| **40** | 100.0 | 93.6 | 94.0 | 0.71 |
| **50** | 100.0 | 92.0 | 92.6 | 0.67 |
| **60** | 100.0 | 90.9 | 91.5 | 0.64 |
| **70** | 100.0 | 89.8 | 90.5 | 0.62 |

**Table S7:** hybrid model scheme showing various predictions of some samples and the final prediction.

| Seq_ID | Meme_cyto | Meme_imp | Meme_sec | Meme_amla | SVM | Final |
|---|---|---|---|---|---|---|
| **>imp_200** | imp_200:1000 | imp_200:1000 | imp_200:1000 | imp_200:1000 | IMP | IMP |
| **>imp_317** | imp_317:1000 | imp_317:4.8 | imp_317:1000 | imp_317:1000 | IMP | imp |
| **>imp_323** | imp_323:1000 | imp_323:4.8 | imp_323:1000 | imp_323:1000 | IMP | imp |
| **>sec_2** | sec_2:1000 | sec_2:1000 | sec_2:1.3e-225 | sec_2:1000 | SEC | sec |
| **>sec_3** | sec_3:1000 | sec_3:1000 | sec_3:4e-240 | sec_3:1000 | SEC | sec |
| **>sec_10** | sec_10:1000 | sec_10:1000 | sec_10:2.9e-224 | sec_10:1000 | SEC | sec |
| **>sec_11** | sec_11:1000 | sec_11:1000 | sec_11:4.2e-225 | sec_11:1000 | SEC | sec |
| **>sec_12** | sec_12:1000 | sec_12:1000 | sec_12:4.8e-208 | sec_12:1000 | SEC | sec |
| **>sec_13** | sec_13:1000 | sec_13:1000 | sec_13:1.9e-225 | sec_13:1000 | SEC | sec |
| **>sec_14** | sec_14:1000 | sec_14:1000 | sec_14:9.6e-238 | sec_14:1000 | SEC | sec |
| **>sec_15** | sec_15:1000 | sec_15:1000 | sec_15:1.1e-159 | sec_15:1000 | SEC | sec |
| **>sec_16** | sec_16:1000 | sec_16:1000 | sec_16:2.7e-155 | sec_16:1000 | SEC | sec |
| **>sec_17** | sec_17:1000 | sec_17:1000 | sec_17:5.6e-167 | sec_17:1000 | SEC | sec |
| **>amla_1** | amla_1:1000 | amla_1:1000 | amla_1:1000 | amla_1:6.5 | CYTO | amla |
| **>amla_2** | amla_2:1000 | amla_2:1000 | amla_2:1000 | amla_2:6.5 | CYTO | amla |
| **>amla_11** | amla_11:1000 | amla_11:1000 | amla_11:1000 | amla_11:3.2 | AMLA | amla |
| **>amla_13** | amla_13:1000 | amla_13:1000 | amla_13:1000 | amla_13:9.7 | IMP | amla |
| **>amla_14** | amla_14:1000 | amla_14:1000 | amla_14:1000 | amla_14:9.7 | IMP | amla |

Where **Meme_cyto**: hits from meme model for cytoplasmic proteins, **Meme_imp**: hits from meme model for integral proteins, **Meme_sec**: hits from meme model for secretory proteins, **Meme_amla**: hits from meme model for membrane attached proteins, **SVM**: prediction by SVM models, **Final**: final decision, **cyto**: cytoplasmic, **imp**: integral membrane protein, **sec**: secretory, **amla**: membrane attached protein, **1000:** in case of no hit a default value of 1000 has been given for e.g. `imp_200:1000`.

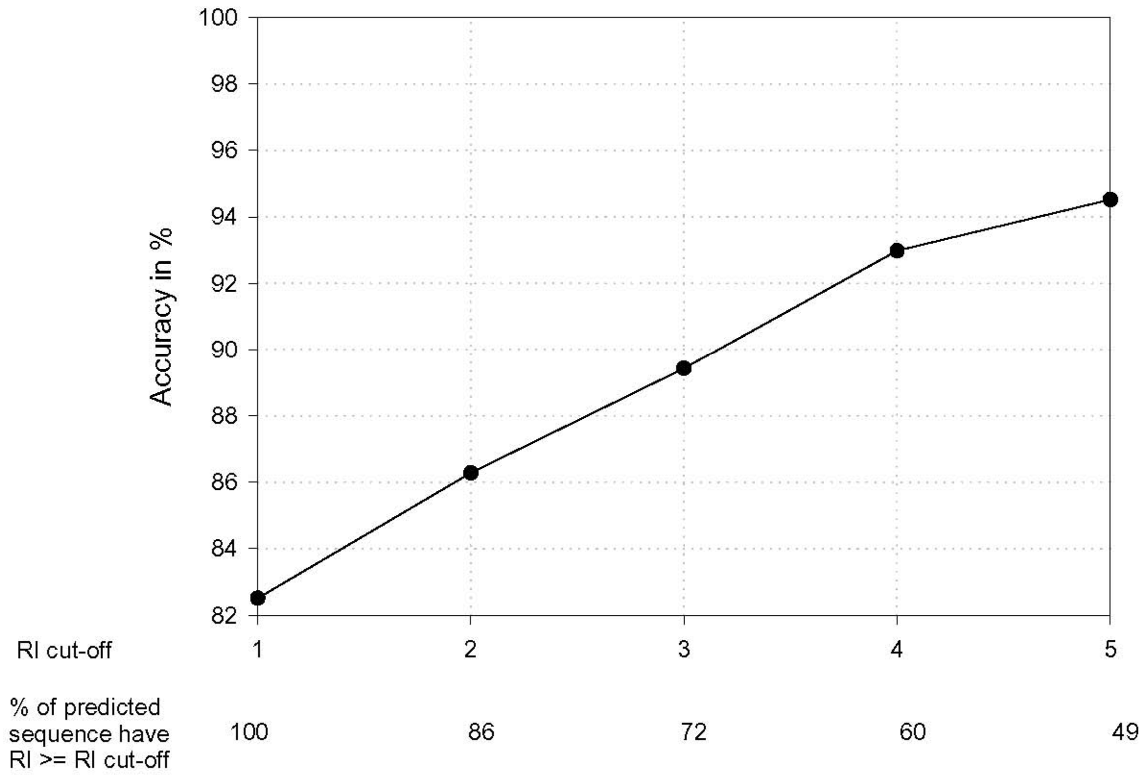


**Figure S1:** Average prediction accuracy with RI >= cut-off value. Percent of predicted sequences having RI >= RI cut-off value are also mentioned. For example, about 72% of sequences having RI >=3 is predicted with about 90 % accuracy, with SVM module using **amino acid composition**, by TBpred server.

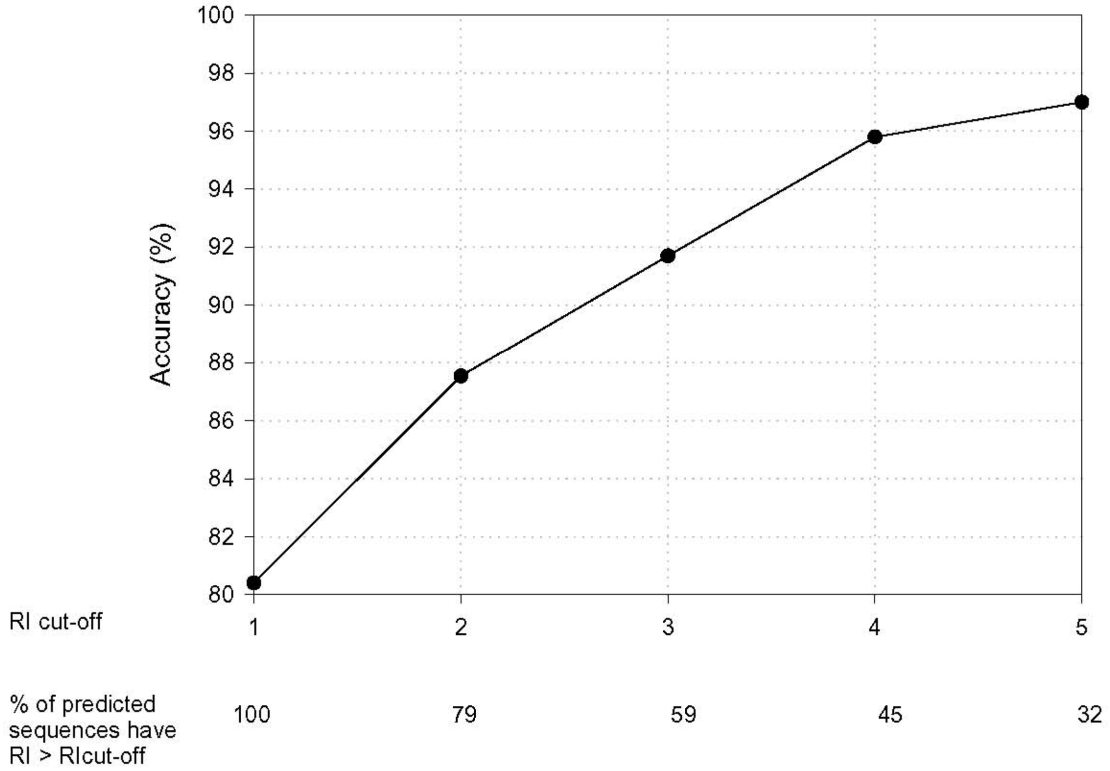


**Figure S2:** Average prediction accuracy with RI >= cut-off value. Percent of predicted sequences having RI >= RI cut-off value are also mentioned. For example, about 60% of sequences having RI >=3 is predicted with 92 % accuracy, with SVM module using **dipeptide composition**, by TBpred server.
